# Abandoned Object Detection in Video-Surveillance: Survey and Comparison

**DOI:** 10.3390/s18124290

**Published:** 2018-12-05

**Authors:** Elena Luna, Juan Carlos San Miguel, Diego Ortego, José María Martínez

**Affiliations:** Video Processing and Understanding Lab, Universidad Autónoma de Madrid, 28049 Madrid, Spain; juancarlos.sanmiguel@uam.es (J.C.S.M.); diego.ortego@uam.es (D.O.); josem.martinez@uam.es (J.M.M.)

**Keywords:** foreground segmentation, stationary object detection, pedestrian detection, abandoned object, survey, video-surveillance

## Abstract

During the last few years, abandoned object detection has emerged as a hot topic in the video-surveillance community. As a consequence, a myriad of systems has been proposed for automatic monitoring of public and private places, while addressing several challenges affecting detection performance. Due to the complexity of these systems, researchers often address independently the different analysis stages such as foreground segmentation, stationary object detection, and abandonment validation. Despite the improvements achieved for each stage, the advances are rarely applied to the full pipeline, and therefore, the impact of each stage of improvement on the overall system performance has not been studied. In this paper, we formalize the framework employed by systems for abandoned object detection and provide an extensive review of state-of-the-art approaches for each stage. We also build a multi-configuration system allowing one to select a range of alternatives for each stage with the objective of determining the combination achieving the best performance. This multi-configuration is made available online to the research community. We perform an extensive evaluation by gathering a heterogeneous dataset from existing data. Such a dataset allows considering multiple and different scenarios, whereas presenting various challenges such as illumination changes, shadows, and a high density of moving objects, unlike existing literature focusing on a few sequences. The experimental results identify the most effective configurations and highlight design choices favoring robustness to errors. Moreover, we validated such an optimal configuration on additional datasets not previously considered. We conclude the paper by discussing open research challenges arising from the experimental comparison.

## 1. Introduction

Developing automated video-surveillance systems is attracting huge interests for monitoring public and private places. As these systems become larger, effectively observing all cameras in a timely manner becomes a challenge, especially for public and crowded places such as airports, buildings, or railway stations. The automatic detection of events of interest is a highly desirable feature of these systems to enable focusing the attention on monitored places potentially at risk.

In the video-surveillance domain, Abandoned Object Detection (AOD) has been thoroughly investigated in the last few years for detecting events of wide interest such as abandoned objects [1] and illegally parked vehicles [2]. AOD systems analyze the moving objects of the scenario with the objective of identifying the stationary ones, which become the candidates to be abandoned objects. Later, a number of filtering steps validate the candidates in order to determine whether they are vehicles, people, or abandoned objects.

AOD systems face several challenges when deployed. They are required to perform correctly under complex scenarios with changing conditions and a high density of moving objects. Many visual factors impact AOD performance such as image noise, appearing in low-quality recordings; illumination changes, either gradual or sudden, camera jitter, and camouflage between a foreground object and the background are some of the challenges in background subtraction approaches. Dynamic backgrounds, containing background moving objects, are also an important issue to be taken into account. Moreover, challenges with processing data in real time emerge as large amounts of data must be handled by the (relatively) complex AOD systems composed of several stages. Another critical challenge concerns the unsupervised operation for long periods of time where the effect of visual factors dramatically decreases performance and errors commonly appear in early stages of AOD systems, which are propagated to the subsequent stages.

Current AOD systems mostly focus on two main stages of the processing pipeline: stationary object detection and classification. The stationary object detection task aims to detect the foreground objects in the scene remaining still after having been previously moving. Once stationary objects are located, the classification task identifies if the static object is an abandoned object or not. Despite the number and variety of proposals, there is a lack of cross-comparisons (both theoretically and experimentally), which makes it difficult to evaluate the progress of recent proposals. In addition, these approaches provide partial solutions for AOD systems, as only one stage of the full pipeline is studied. The impact of these partial solutions is rarely studied for larger end-to-end systems whose input is the video sequence and the output is the abandoned object event. Moreover, existing experimental validations are generally limited to few, short, or low-complexity videos. Therefore, system parameters may be over-fitted to the specific challenges appearing in the small datasets, which makes it difficult to extrapolate conclusions to unseen data (e.g., long-term operation).

To address the above-mentioned shortcomings, this paper proposes a canonical framework representing the common functionality of AOD systems and surveys each stage of these systems. We critically analyze recent advances for moving and stationary object detection, people detection, and abandonment verification applied to AOD systems. We also provide experimental comparisons for traditional and recent approaches. A multi-configurable AOD system (software available at http://www-vpu.eps.uam.es/publications/AODsurvey/) is created to choose among different alternatives for each stage, thus enabling one to generate a large range of AOD systems with different combinations. Such a multi-configurable system enables one to study deeply the trade-off between the accuracy and computational cost of AOD systems.

The paper is organized as follows. Section 2 compares related surveys. Section 3 overviews the common framework for AOD systems, whereas Section 4 discusses existing approaches for each stage. Section 5 defines the experimental methodology, and Section 6 presents the experimental results. Finally, Section 7 concludes this paper.

## 2. Comparison to Other Surveys

Table 1 compares recent surveys for automated video-surveillance. This paper complements other surveys for AOD systems regarding moving object detection [3,4,5], stationary object detection [6,7], people detection [8,9], activity recognition [10,11], abnormal behavior recognition [12,13], and multi-camera analysis [14,15]. Most surveys are focused on providing a deep coverage for moving object detection and behavior recognition. However, these surveys superficially deal with events such as abandoned object detection or illegally-parked vehicles. Moreover, existing datasets are frequently analyzed, but experimental comparisons are rarely provided, which limits the conclusions that can be drawn from such analysis. Albeit that our proposal may share some similarities with some surveys discussing stationary foreground detection [6,7] (a key stage for AOD systems), this paper analyzes all stages in the processing pipeline of AOD systems including critical reviews of state-of-the-art approaches. We improve [7] by proposing a new taxonomy for stationary foreground detection based on concepts that do not overlap across categories, as in [7] (e.g., persistence is shared by several categories). Recently, [7] surveyed Stationary Foreground Detection (SFD), organizing the literature into seven categories (tracking, persistence, dual background, classifiers, Gaussian stability, combinations, and others) and denoting robustness or not against five properties (occlusions, long-term, stationary pedestrians, removed or stolen object, and ghost regions). This classification, though exhaustive, defines categories with concepts that are not unique to each category. For instance, persistence is used to detect static objects that are later verified via tracking or classifiers, and dual background models are based on persistence to determine stationarity. Therefore, we prefer to adopt a different perspective that focuses on reviewing the ideas behind the algorithms for SFD, providing a panoramic view of them and their evolution in time rather than an exhaustive survey of every published approach. Additionally, we provide experiments (see Section 6) to reveal insights into good stationary foreground detectors. Moreover, only one survey provided experimental comparisons for one stage of AOD systems [6], though limited to a small set of sequences. This paper studies the performance impact of different alternatives for each stage, a key contribution that is not provided by any compared surveys. Finally, software implementations are made available online to the research community to foster comparisons with new proposals.

## 3. Canonical Framework

**Figure 1:**
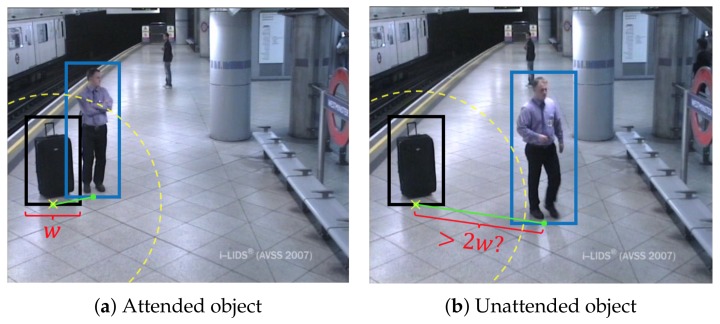
Example of abandoned luggage for the AVSS_AB_2007 sequence (http://www.eecs.qmul.ac.uk/~andrea/avss2007_d.html). Subfigure (**a**) shows an abandoned object when it is attended. An object (black box) is considered attended if a person (blue box) lies within a distance of twice the object’s width radius. In subfigure (**b**) the object is unattended since the person moves away farther than the defined distance.

Abandoned objects can be determined by two rules: the candidate object is stationary and unattended. The former defines a temporal rule where an object is considered as stationary if it has remained without moving for a certain period of time, which depends on the application, being usually 30 or 60 s. The latter corresponds to a spatial rule where an object is considered as unattended if the object owner (i.e., the person that left the object) is not spatially close to the object. Such closeness is often defined by considering an ellipse or circle whose radius is proportional to the object size (e.g., often set to three-times the object width or a fixed value of 3 m [19]). Both rules have to be fulfilled in order to consider an abandoned object event, as depicted in Figure 1.

**Figure 2:**
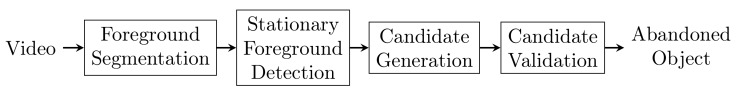
Canonical framework for abandoned object detection.

The frameworks of AOD systems proposed in the literature can be unified using the diagram of Figure 2. This diagram consists of several stages for foreground segmentation (i.e., detect the regions of interest or blobs), stationary foreground detection (i.e., determine which ones do not move for a certain period), candidate generation (i.e., identifying the objects potentially being abandoned), and candidate validation (i.e., deciding whether the objects are abandoned or not). Existing proposals for abandoned object detection may partially implement the above-mentioned definition of abandoned objects by using only the first and second stages for the temporal rule; and the third and fourth stages for the spatial rule. As the performance of each stage relies on the previous one, AOD research is often directed towards the first and second stages.

## 4. Stages of Abandoned Object Detection

In this section, we survey the literature for each stage of the abandoned object detection systems.

### 4.1. Foreground Segmentation

Foreground segmentation is key in many applications, such as video-surveillance [20], aiming to classify each image pixel into foreground or background, thus producing a foreground mask containing the regions of interest (i.e., blobs), which represent the foreground [20,21,22]. For example, such a foreground can be related to every object in the scene [23,24] or only to the salient objects [21]. In videos, the foreground may correspond to all moving objects [20], to specific temporal salient objects [25], to some relevant spatio-temporal patterns [26], or to pre-defined image labels [27].

Background Subtraction (BS) is often used for AOD due to the relative control of camera motion. Traditional BS algorithms usually consist of four stages [20]: modeling, to statistically represent the background of the scene; initialization, to acquire the first model; maintenance, to adapt the model to scene variations over time; and detection, to segment foreground objects by comparing each frame and the model. Segmenting the foreground addresses several challenges affecting segmentation performance [20]. False positives may be caused by illumination changes (non-accurate model adaptation), camera jitter (pixel misalignment between current and background images due to camera motion), ghosts (objects wrongly included in the background model), dynamic backgrounds (background motion difficult to handle by the model), camouflages (foreground and background sharing similar appearance), and cast shadows (shadows from objects are sometimes detected). A high variety of approaches is proposed to overcome these challenges, which can be classified by the type of model employed: Gaussian and support vector models [28,29], non-parametric models [30,31,32], subspace learning models [33,34], neural networks [35,36], and RPCA (Robust Principal Component Analysis) and sparse models [37]. These models can also use different features (or combinations thereof) such as color, gradient, texture, and motion [38]. Moreover, deep learning models [39,40] have recently emerged as promising frameworks to unify modeling and feature selection. However, these models are limited to employing training and test data from the same video sequence.

**Figure 3:**
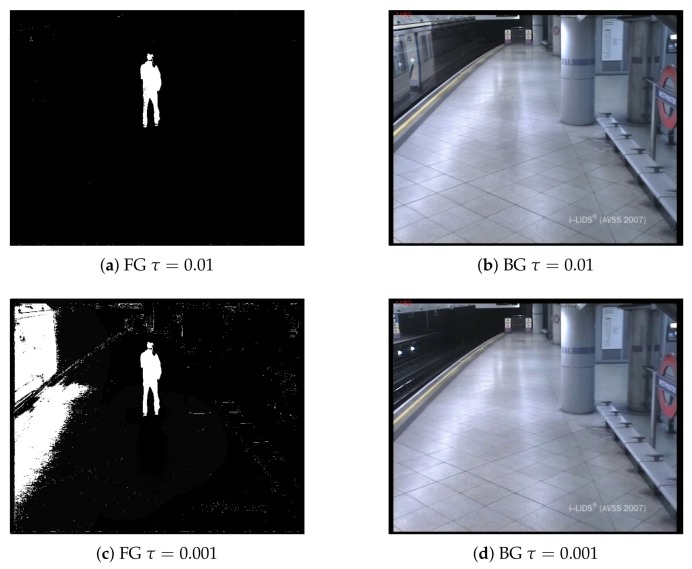
Examples of different adaptation rates for background subtraction based on the Mixture of Gaussians approach [45] for Frame 1180 of the sequence AVSS_AB_2007 (http://www.eecs.qmul.ac.uk/~andrea/avss2007_d.html). Subfigures (**a**,**c**) show Foreground (FG) masks with different parameter τ, which controls the update rate of the Background (BG) models, that are depicted in subfigures (**b**,**d**).

AOD state-of-the-art systems [2,41,42,43] widely employ Mixture of Gaussian (MoG) [28] and Kernel Density Estimation (KDE) [30] approaches for BS. This choice might not seem optimal, as recent BS approaches clearly outperform MoG and KDE according to recent benchmarks [44] such as the Pixel-based Adaptive Word Consensus Segmenter (PAWCS) approach [32]. However, such improvement is mostly achieved by removing false positives at the cost of increasing false negatives. Recent approaches increase the update rates of the background models [44], so scene changes (e.g., illumination and shadows) are quickly incorporated into the model. Figure 3 shows examples of different update rates and the effect on the foreground segmentation masks for abandoned objects. Other approaches propose complex strategies requiring many parameters [32], which are difficult to adjust for different scenarios of AOD. These strategies increase AOD challenges and might lead to missing AOD events. By changing the rate of the update scheme, stationary objects may be quickly incorporated into the background models, thus becoming false negatives. Therefore, BS approaches exhibit a trade-off between adaptability of models to changes and the capability of detecting stationary foreground. Furthermore, better algorithms often require additional computations, and therefore, lower frame-rates can be achieved. Hence, unlike initially thought, simple algorithms might be a good choice as they are fast and their limitations may be palliated by the remaining AOD stages, which involve the removal of false positives using temporal information and image properties. We analyze this apparent contradiction in the experimental work by analyzing the effect of BS approaches in the AOD performance (see Section 6) and demonstrating that classical approaches [28,30] behave in a similar way to recent top-performing BS approaches in this context, but requiring a significantly lower computational cost.

### 4.2. Static Foreground Detection

Stationary Foreground Detection (SFD) aims to detect objects that remain in place for some time in a video sequence by analyzing spatio-temporal persistent patterns. Existing approaches focus on extracting such patterns from background subtraction approaches [46,47,48] or other features [49,50]. We organize the literature into the following categories (see Table 2 for a summary of the main approaches): single foreground mask, multiple foreground mask, model stability, and other approaches. The three first categories represent the main literature in the field, whereas the last category includes methods of a different nature that do not have an independent category due to their uniqueness. Note that we focus on static camera scenarios, but there are also some approaches dealing with moving cameras [51,52,53,54,55].

#### 4.2.1. Single Foreground Mask

**Figure 4:**
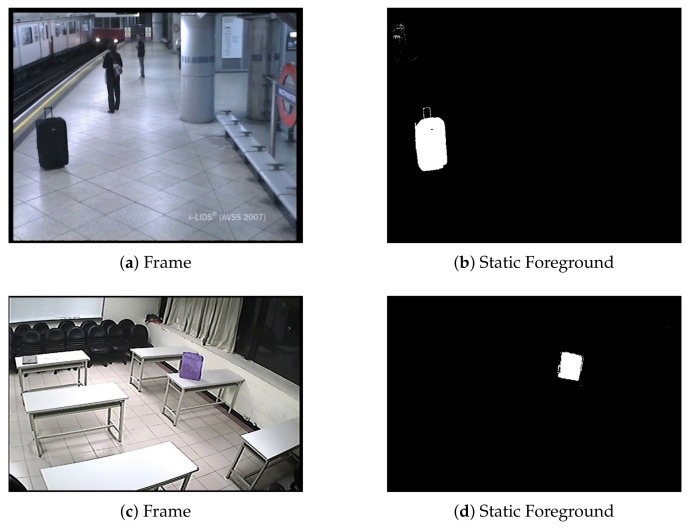
Static foreground detection computation using a simple persistence approach for Frame 3200 of the sequence in AVSS_AB_easy_2007 (http://www.eecs.qmul.ac.uk/~andrea/avss2007_d.html) and Frame 2000 of the sequence ABODA_video3 (http://imp.iis.sinica.edu.tw/ABODA). Subfigures (**a**,**c**) show both image frames, and their respective static foreground masks are depicted in subfigures (**b**,**d**).

This category comprises approaches that use the foreground mask of a background subtraction algorithm as the base for the persistence analysis. The main approach par excellence is the accumulation of foreground segmentation masks [63] that computes a staticness map $F\in \left[0,1\right]$ by:

(1) $$F_{t}^{p}=\left\{\begin{array}{cc} F_{t-1}^{p}+\alpha , & if\;M_{t}^{p}=1\\ F_{t-1}^{p}-\beta , & if\;M_{t}^{p}=0 \end{array}\right.,$$

where each pixel p in F is increased (decreased) by α (β) when foreground (background) is detected in the foreground segmentation mask M, α is adjusted to reach one when the user-defined alarm time *T* is completed, and β should be high to allow fast decreases of F values when the background is detected (though in practice, a high value may lead to false negatives due to the camouflage of occluding objects). Therefore, when the foreground is detected, the staticness increases with time. Then, F is a spatio-temporal modeling of the persistence; thus, thresholding this map leads to a stationary foreground mask (usually, the threshold is close to one). Figure 4 depicts examples of stationary foreground masks obtained with a persistence approach.

In [64], the foreground mask is post-processed analyzing blob contours and structural properties to obtain an improved mask that is later accumulated and thresholded to obtain a stationary foreground mask. Then, a people detector is run over the blobs of the stationary foreground mask to determine the abandoned objects. More recently, in [65], foreground accumulation was seen as a time filter and applied together with additional filters that checked certain geometrical and appearance properties over foreground blobs. Instead of sequential filters, the same authors proposed in [48] to analyze stationary blob candidates, obtained via foreground accumulation and thresholding, by jointly learning an SVM model from intensity, motion, and shape features to classify the candidates between abandoned or removed. Again, these authors proposed further SFD algorithms [56,66,67] based on intensity, motion, shape, and foreground accumulation features to learn the transitions between the states of three FSMs that model the spatio-temporal patterns at different abstraction levels (pixel, blob, and event) to detect abandoned and stolen objects. Moreover, the accumulation of foreground and motion features with occlusion handling was proposed in [68] and extended in [57] with structural features to increase robustness against illumination changes before thresholding a stationary history image (the staticness map). Furthermore, there are several approaches [69,70,71,72] adopting different complexities of object tracking over blobs extracted from a stationary foreground mask [63]. These approaches may introduce filters as previous approaches, but their main characteristic is that they introduce robustness against occlusions and illumination changes through object tracking, which allows the verification of the same stationary object being accumulated over time. Instead of tracking, Ref. [73] used a self-organizing model for background subtraction and, once the stationary foreground was computed by accumulation and thresholding, also to validate the persistence of stationary objects over time. Note that modeling the stationarity also enables a precise knowledge of the lifetime of a stationarity object, which is needed to trigger an alarm precisely when the alarm time is reached.

Also based on single foreground masks, some approaches subsample a few temporal instants over time to check stationarity from either the foreground mask alone [74] or together with motion information [75], thus avoiding a continuous accumulation in each video frame. However, though fast, this approach does not properly deal with occlusions, thus being limited to simple scenarios. More recently, in [58], background subtraction and frame difference between non-continuous frames were used to determine stationary candidates. Then, a cascade of deep learning classifiers was applied to verify the object type (luggage) and the attendance or not of the object.

#### 4.2.2. Multiple Foreground Masks

Dual background models were originally proposed in [76,77] to detect stationary objects from two background subtraction models that were updated at different learning rates, the so-called short- and long-term background models. The fact that they used two models led them to consider more complex spatio-temporal patterns, defining four possible states for pixels in the scene: moving object, candidate abandoned object, uncovered background, and background. These states are inferred by comparing the short- ($M_{t,S}$) and long-term ($M_{t,L}$) foreground masks:

(2) $$State=\left\{\begin{array}{cc} Moving, & if\;M_{t,S}=1\;\wedge \;M_{t,L}=1\\ Stationary, & if\;M_{t,S}=0\;\wedge \;M_{t,L}=1\\ Uncovered, & if\;M_{t,S}=1\;\wedge \;M_{t,L}=0\\ Background, & if\;M_{t,S}=0\;\wedge \;M_{t,L}=0 \end{array}\right.,$$

where all pixels classified as *Stationary* are used to obtain a stationary foreground mask by accumulation and thresholding as done in [63].

The dual background approach has been widely used in the literature over the years [41,77,78], adding different functionalities to improve performance. In [79], some heuristics and a simple block tracker based on spatial location features were added to model persistence while handling occlusions. Furthermore, in [80], short- and long-term foreground masks were post-processed using structure features to increase robustness against illumination changes, and once the stationary objects were detected, a HOG-based people detector was used to remove stationary pedestrians. Furthermore, dual background models have been extended to consider more complex spatio-temporal patterns by modeling the transitions between the four states [46] (see Equation (2)) or an extended set of states [81] (several of the states denote stationarity) through an FSM. Going back to the original dual background model, in [82], this was applied in the YUV color space instead of the RGB to increase robustness against illumination variations, and stationary objects were verified to be abandoned or stolen via image contours. Moreover, in [60,83], backtracking was proposed to verify the location of owners compared to the stationary candidate, using the stationary objects computed by a standard dual background model. More recently, the authors of [1,2] applied dual background modeling for, respectively, abandoned object detection and illegal parked vehicle detection. The former is based on comparing the short- and long-term background images to compute a stationary foreground mask whose evolution at the pixel level is used to cluster the detection patterns into four states: static-to-background, background-to-static, background, and noise; using the background-to-static state pixels to extract blobs that are later filtered with temporal constraints that check the alarm time, a people detector to discard pedestrian detectors, and a proximity rule to verify that object owners left their belongings. The latter applies a standard dual background model followed by a set of filters to verify geometrical properties, distinguish vehicles from any other object, and track them to improve the persistence modeling. Recently, the dual background model was extended to a triple background model [59] by including a medium-term model where, following the same ideas of [46], an FSM and accumulation and thresholding were used to report the final alarms.

#### 4.2.3. Model Stability

Another widely-used approach to detect stationarity is to consider the stability of the different modes of a multi-layer background subtraction method (typically, the Gaussians of a Mixture of Gaussians). Initially, this approach was proposed in [84] introducing several recurrent concepts in approaches focusing on this view. First, each pixel was modeled with a mixture of three Gaussians where the weights of each one revealed the nature of the pixels belonging to that Gaussian: background (highest weight), static (medium weight), and foreground (lower weight). Note that Gaussian learning rates needed to be adjusted to respect an alarm time. Then, structural and luminance features were used to deal with illumination changes and shadows. One important issue of this approach is the healing problem, i.e., the different rates of absorption of objects across different Gaussians. To cope with this issue, the authors detected the moment of the maximum size of a stationary object to force that region to push back to the background Gaussian. Additionally, this approach analyzes edges in the scene to classify stationary objects as abandoned or stolen. The authors evolved this algorithm in [85] to deal with different frame-rates and still respecting the alarm time that was determined by the persistence computed by a template-matching tracking of the healed regions (regions pushed back to the background). Furthermore, the authors introduced a region-growing technique for abandoned and stolen detection to address the limitations of [84] with occlusions when classifying abandoned and stolen objects. The authors further improved the algorithm [62] by introducing a history of background objects to prevent ghost artifacts and three people detectors to deal with different camera views, and they also exploited the tracking trajectories to assure that stationary objects intersected them.

Moreover, this type of approach was exploited in four works of the same authors [47,61,86,87]. Firstly, in [86], they built on [84] by adopting the three Gaussians, but they introduced an FSM to model static pixels once they were detected in the static Gaussian, thus increasing the complexity of the spatio-temporal patterns modeled. Then, they also validated each detection by adopting similar foreground features as those presented in [64] and the region growing and template matching from [85]. Secondly, the authors further improved their results in [87] by avoiding the updating of the background Gaussian in a foreground region and by slowing down the updating of the static Gaussian and the foreground Gaussian to increase, respectively, robustness against foreground-background blending, ghost artifacts, and occlusions. Additionally, they extended the foregroundness validation by learning an SVM model from multiple features, thus reducing the false positives. Extending this approach, in [47], they used their initial approach [86] and improved the candidate validation by using a relative attributes framework that allowed them to rank the alarms, thus reporting the true alarms as the top ranked ones. Moreover, the authors proposed an illegally-parked vehicle detector [61] building on [86] and adding a FAST keypoints-based tracker to verify persistence, while being robust to typical occlusions among cars.

More recently, in [88], model stability was exploited for long-term video analysis. The algorithm works in a block-wise manner and uses a fast online clustering robust to illumination changes to associate spatio-temporal changes in the most stable clusters with stationary objects.

#### 4.2.4. Others

There are some approaches for SFD that do not lie in previous categories. Therefore, we have created this category to compile some additional relevant methods. In [49], illegally-parked vehicles were detected using Harris keypoints (stable keypoints from the previous day of a given scenario are assumed to be the background and discarded in the current day) to create spatio-temporal maps that allow the detection of persistent objects in the scene. The method focuses on keypoints and not on trying to detect and track vehicles, thus avoiding the difficult detection of cars with different sizes and appearances. However, the recent success of object detectors based on deep learning techniques has enabled solving that issue. For example, in [89], illegally-parked vehicles were detected using the object detector SSD (Single Shot MultiBox Detector) [90], where aspect ratios of bounding boxes were modified to meet the scenario conditions. Then, tracking through template matching was performed to analyze the persistence of detected objects in the same spatial location. Moreover, in [91], Independent Component Analysis (ICA) was proposed for the analysis of spatio-temporal persistent patterns through the *t*-statistic (which is expected to be similar to a step function for abandoned objects) in gray-scale videos. Furthermore, this approach was extended to consider color videos by moving from ICA to Independent Vector Analysis (IVA) in mono-camera [92] and multi-camera scenarios [93]. Recently, [50] proposed to overcome the limitations of approaches based on pixel intensities by analyzing persistent foreground edges, which were clustered to obtain a stable edges mask and analyzed in terms of position and stability in time to delineate the object bounding box. Furthermore, an abandoned object classifier based on edges’ position, orientation, and staticness scores was applied.

### 4.3. Candidate Generation

**Figure 5:**
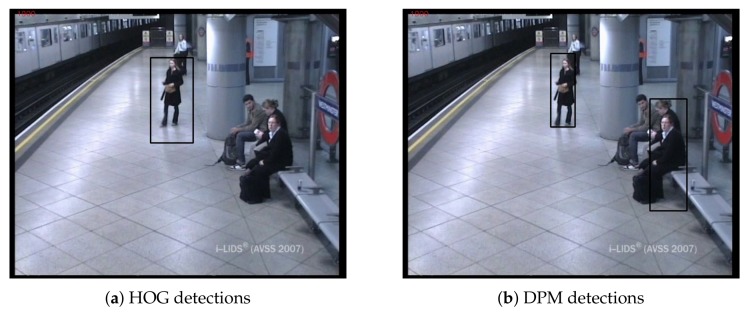
People detection results using HOG [95] (**a**) and Deformable Part Model (DPM) [96] (**b**) algorithms in AVSS AB 2007 http://www.eecs.qmul.ac.uk/~andrea/avss2007_d.html. As we can observe, the holistic HOG model is not able to detect the woman sitting, while the part-based DPM detects her, although it fails at detecting the other sitting people.

Depending on the final application, static objects of interest, i.e., candidates of interest, may vary. There are works only focused on detecting abandoned luggage [50,94]; others are geared towards illegally-parked vehicle detection [2,49]; and others adopt a comprehensive approach [60,78]. Since detecting abandoned objects, in general, is the goal of interest, one can make the assumption that whatever is not a person can be considered as an object. Following this strategy, this distinction problem between people and objects can be solved simply by applying a people detector. Figure 5 illustrates an example of people detection results over two sample frames.

People detection surveys providing an exhaustive study of the existing conventional techniques based on hand-crafted feature-based models are available in the literature [9,97]. Let us briefly classify and summarize, regarding the characterization of the person model, the available algorithms into three main groups. Motion-based approaches only use information with respect to the person movements; thus, they are not able to deal with partial occlusions. The authors of [98] proposed a system based on periodic motion analysis including tracking to increase robustness, while [99] suggested a detection system based on detecting people motion patterns. Appearance-based approaches exploit people appearance information such as color information, silhouettes, or edges. They can be divided into holistic and part-based models. Holistic models, such as [95,100,101], are simple models representing the human body as a whole. They cannot deal with partial occlusions, nor people pose variations. In contrast, part-based models, e.g., [96,102,103,104], are more complex models defining the body as a combination of multiple regions, and they can detect partially-occluded people and people with different poses. The last category combines and takes advantages of both motion and appearance. Table 3 summarizes the robustness of the categories previously mentioned.

However, the last few years have shown huge progress in pedestrian detection due to deep learning methods’ emergence [105,106,107]. Both objects and people detector approaches based on Convolutional Neural Networks (CNN) are able to learn features from raw pixels, outperforming models based on hand-crafted features. Let us arrange learning methods in the following way. Two-stage approaches first compute region proposal methods over the input to compute potential bounding boxes that are secondly classified. R-CNN framework approaches [108,109,110] are currently the top detection two-stage methods. On the other hand, single-stage approaches reframe the two stages (region proposal and classification) at a single-stage regression problem, being less time-consuming. Four state-of-the-art single-stage models are SqueezeDet+ [111], You Only Look Once (YOLOv2) [112], Single Shot MultiBox Detector (SSD) [90], and Deconvolutional Single Shot Detector (DSSD) [113]. Recent work in [114] proposed closing the gap between single- and two-stage approaches using a single loss function sensitive to already learned examples.

**Figure 6:**
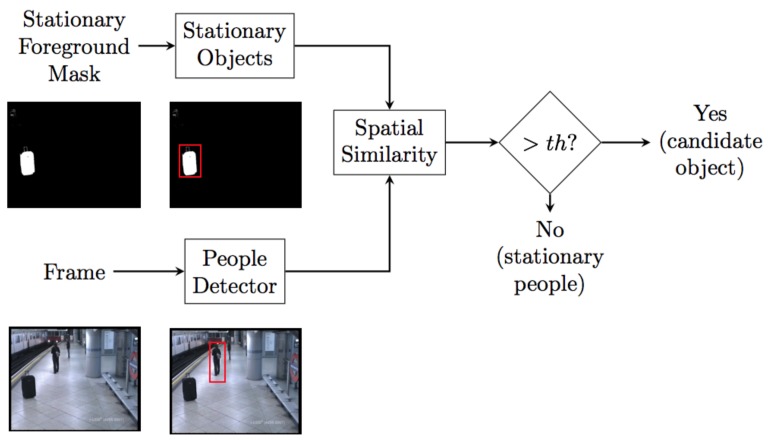
Block diagram of the candidate generation module. Stationary objects and people detection blobs are independently extracted and then spatially compared. If a stationary object overlaps with people detection, it is considered as a static person, otherwise the object is a potential abandoned object candidate.

Several state-of-the-art abandoned objects detection systems do not include a people detection stage, since they do not consider false detections caused by still people or they simply accept them. Alternatively, other works do incorporate a people detection stage for candidate classification. The Haar-like features full-body classifier, described in [115], is a classifier based on a trained holistic person model; thus, it is very efficient. It is used in the proposed abandoned object detection system in [43]. The deformable part-based model, proposed in [96], is a part-based person model, which was used in [60] for people detection. Depending on the purpose, candidates can be restricted to an object category, such as cars; therefore, a specific detector/classifier is required. The Histogram of Oriented Gradients (HOG) applies exhaustive search based on appearance descriptors along the whole image. It was used for car detection in [2], although it was initially proposed in [95] for human detection. All previously-mentioned technologies were appearance-based, but this can also be combined with tracking for people detection, e.g., [62]. Figure 6 shows a block diagram of this stage, where its operation is illustrated.

### 4.4. Candidate Validation

**Figure 7:**
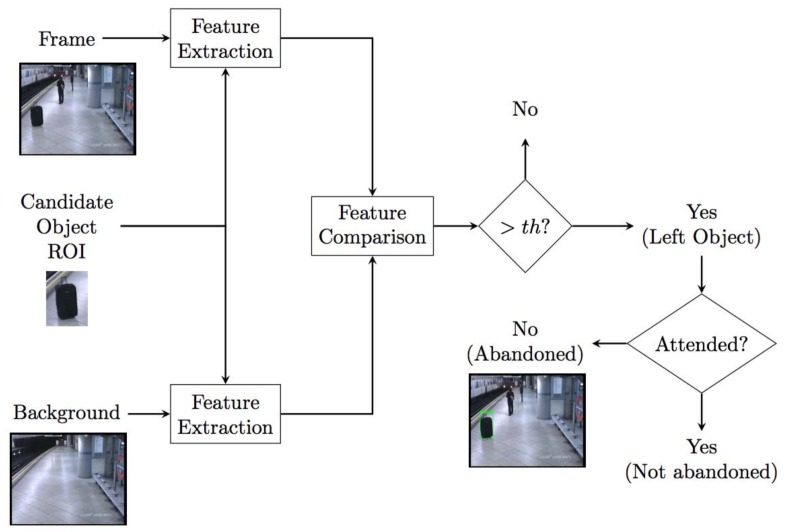
Block diagram of candidate validation module. In simple terms, for each candidate, its region of interest is extracted, and certain features are extracted comparing them with the background and current frame. Through this comparison, false objects are discarded. Finally it is checked if the object is attended.

Once the static foreground has been classified, object candidates can be known. The last stage in abandoned object detection systems consists of validating the candidates. This stage is necessary to discard false detections due to illumination changes or removed objects, for instance. This validation stage includes two differentiated sub-stages: left-object validation and unattended validation. Left-object validation checks candidate’s nature in order to remove false positives due to illumination changes or removed objects. Once it is verified that the candidate is an abandoned object, it is important to check its surroundings, looking for potential owners in order to verify that the object is, indeed, unattended. A block diagram presenting the functioning scheme of this stage is shown in Figure 7.

#### 4.4.1. Left-Object Validation

Analyzing the candidate’s origin is necessary to discard static objects not due to abandonment. Static object classifiers allow us to perform this task. A concise review of them is provided in [116]. As a brief summary, according to the features they employ, they can be classified into the following categories. Edge-based approaches consider the energy of the static object region boundaries and make the comparison with the same region in the background image. Since they are not affected by pixel colors, they are robust to camouflage. Color-based approaches analyze the color information of the internal and external regions demarcated by the bounding box and the boundaries of the detected static object; thus, they are not able to deal with the camouflage challenge. Lastly, hybrid approaches, as a combination of the previous methods, combine edges and color information in order to determine the object nature. In addition to these classifiers, shape and size filtering can be applied to remove false positives. Noise false positives can be removed by filtering candidates by a predefined minimum size [2,42,94]. The aspect ratio is a useful filtering feature when detecting a specific type of object, such as cars, in [2].

**Figure 8:**
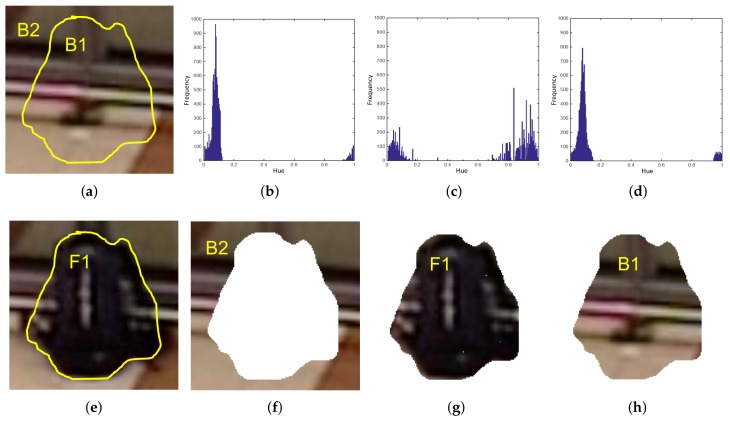
Example of Pixel Color Contrast algorithm operation. Subfigure (**a**) shows initial frame and (**e**) depicts an abandoned bag in a posterior frame. PCC computes Hue histograms, (**b**–**d**), from B2 (**f**) , F1 (**g**) and B1 (**h**), respectively. Histogram comparisons (**b**,**c**) and (**c**,**d**) are made and final abandoned decision is taken from the results. Since (**b**) is more similar to (**d**) than to (**c**), this means (**e**) represents an abandoned object.

State-of-the-art proposals for abandoned object detection employ different techniques for candidates’ classification. Color-based approaches were used in the systems proposed in [43,117]; however, hybrid approaches are the most widespread. In [118], the authors proposed a complete abandoned object detection system using a fusion hybrid approach to classify potential candidates. Another innovative method also combining edge and color information, called Pixel Color Contrast (PCC), was proposed in [119] for this aim. Figure 8 illustrates an example of the color-based approach proposed in [119].

#### 4.4.2. Unattended Validation

When a static object candidate is finally identified as abandoned, it is also important to analyze its surroundings to check if the object is being attended by someone. Not all state-of-the-art works address this problem in the same way. There are two prevailing approaches coping with this problem in the recent literature.

The first strategy does not consider whether the object is attended or not. These kinds of works do not analyze the object surroundings [43,50,56]; thus, they do not consider the possibility of the object being attended by a person. Therefore, they avoid evaluating video sequences containing this scenario, or they simply cope with those errors. A disadvantage of this strategy is the fact that every static object will be detected, no matter whether it is someone’s property; thus, many false detections will be generated. On the other hand, it requires less computational cost since it is a simpler approach.

The second strategy does differentiate between attended and unattended objects, and only unattended objects will be considered as abandoned. They do not contemplate owner identity identification, but they establish a proximity rule [42,60]. The closest person within a radius centered in the abandoned object will be considered as the owner attending the object; this way, the system will not identify it as abandoned. The main advantage of this strategy is the considerable reduction of false detections; however, failures at the people detector can lead to a missed detection.

### 4.5. Available Datasets

This section introduces the most used and significant datasets for abandoned objects in the literature. In particular, those that are employed in the video-surveillance scope. Table 4 lists each dataset along with the number of sequences, the average sequence length, its scenario, and the challenges.

## 5. Experimental Methodology

We propose a multi-configuration system (Software available at http://www-vpu.eps.uam.es/publications/AODsurvey/) for systematically evaluating existing approaches for each stage of the canonical AOD system. In this section, we list the selected approaches to be employed for each stage. Then, we cover the selected datasets and the employed evaluation metrics.

### 5.1. Selected Approaches

For the foreground segmentation stage, we have considered MoG [28] as the baseline, since it is widely used across the AOD literature, and we have also included alternative approaches not employed within the context of AOD systems, such as KNN and PAWCS. K-nearest neighbors background subtraction (KNN) [45] was included because it is a simple and fast improved version of MoG. The Pixel-based Adaptive Word Consensus Segmenter (PAWCS) [32] was selected as the top-performing proposal according to the evaluations of CDNet2012 and 2014 [120].

For the Static foreground detection stage, we have included the most common approach employing two background models (DBM) [77] and the recent extension to three models (TBM) [59]. Moreover, we also have integrated the foreground accumulation approach (ACC) [63]. For sub-sampling approaches, we have included the temporal multiplication of foreground masks (SUB) [121]. The last included approach is multi-feature combination based on the history images of three features (foreground, motion, and structural information) (MHI) [57]. Note that we do not include any tracking-based approach, as they are better suited for low-density scenarios and have been clearly outperformed, according to published results [118].

For the candidate generation stage, we apply a people detector to generate potential candidate objects from the static foreground regions. Therefore, we term this stage as People Detector (PD) in the rest of the paper. We include traditional hand-crafted feature-based approaches already employed in AOD systems such as the Histogram of Oriented Gradients (HOG) [95], the Haar-like feature classifier for full (HaarF) and upper body parts (HaarU) [115] and the Deformable Part Models (DPM) [96]. Moreover, we have also integrated the recent Aggregated Channel Feature (ACF) [122] detector, although it has not been used in this scope, because it has been shown that it outperforms the previously- cited ones [9]. In addition, we have considered as well two neural network-based object/pedestrian detectors, Faster R-CNN [110] and YOLOv2 [112].

For discriminating candidates in the candidate validation stage, we employ approaches to determine if the object is abandoned or not. Therefore, we term this stage as Abandoned Discriminator (AD) in the rest of the paper. We analyze the contour of the candidate based on High Gradients (HG) [118], Color Histograms (CH) [118], and color contrasts (PCC) [119]. For validating the existence of people attending the object in a spatial vicinity, we apply the spatial rule depicted in Figure 1.

### 5.2. Datasets

For evaluation, we selected 21 sequences from the following public datasets: AVSS_AB 2007 (http://www.eecs.qmul.ac.uk/~andrea/avss2007_d.html), PETS2006 (http://www.cvg.reading.ac.uk/PETS2006/data.html), PETS 2007 (http://www.cvg.reading.ac.uk/PETS2007/data.html), ABODA (http://imp.iis.sinica.edu.tw/ABODA/index.html), and VISOR (http://www.openvisor.org/video_videosInCategory.asp?idcategory=12). This selection was carried out according to the definition in Section 3; in essence, the object must be unattended at least for 30 s. Several sequence in the literature do not fulfill the temporal requirement, such as the CANDELA (http://www.multitel.be/image/research-development/research-projects/candela/abandon-scenario.php) dataset and most of the ABODA (http://imp.iis.sinica.edu.tw/ABODA/index.html) sequences. For this reason, they have not been evaluated in this work.

**Figure 9:**
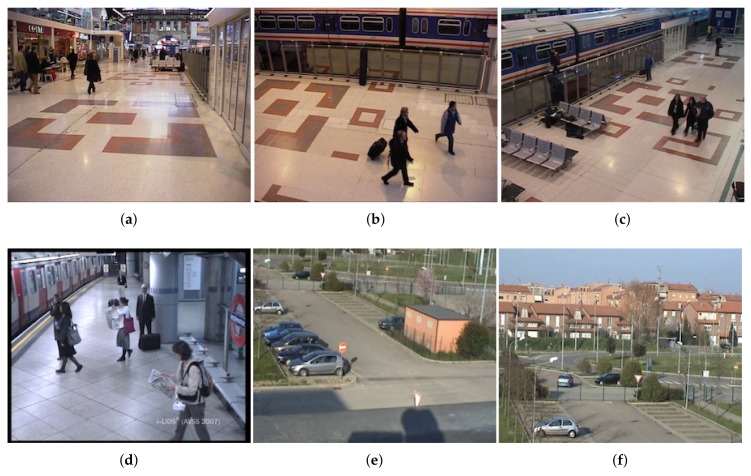
Sample frames from: (**a**) PETS 2006 camera 1, (**b**) PETS 2006 camera 3, (**c**) PETS 2006 camera 4, (**d**) AVSS AB 2007, (**e**) VISOR View 1, and (**f**) VISOR View 2.

The AVSS_AB dataset presents an abandoned luggage scenario with different grades of complexity such as crowds, occlusions, and stationary people. From PETS, we have evaluated three different points of view of the abandoned object scenario. VISOR sequences show a stopped vehicle scenario with two different points of view, and from ABODA, three different indoor and outdoor scenarios have been considered. Figure 9 shows representative frames of the selected sequences.

We have manually annotated the temporal information of the abandoned objects (ground-truth annotations and links to video files available at http://www-vpu.eps.uam.es/publications/AODsurvey/) (i.e., for each object, the frames for starting, ending, and becoming abandoned) in the video sequences using the ViPER-GT (http://www.viper-toolkit.sourceforge.net/) tool according to the abandoned object definition (see Section 3). Table 5 summarizes the temporal information of each abandoned object, in terms of its lifespan in seconds, as well as information about whether the object is unattended or not.

### 5.3. Evaluation Metrics

To evaluate the performance, we compare results with the ground-truth for each sequence by searching for spatio-temporal matches between targets (ground-truth) and candidates (results). For each candidate object $o_{j}$ and ground-truth annotation $g_{i}$, two attributes are considered: its location, in terms of a bounding box, and its lifespan, in terms of start and end frame. Matching is evaluated according to the dice coefficient metric [123], which is defined as follows:

(3) $$Matching\left(o_{j},g_{i}\right)=\left\{\begin{array}{cc} True & \;\mathrm{if}\;dice_{temp}\left(o_{j},g_{i}\right)<\sigma \;\mathrm{and}\;\\ \; & \;dice_{spatial}\left(o_{j},g_{i}\right)<\tau \\ False & \;\mathrm{otherwise} \end{array}\right.,$$

where σ is a threshold for the minimum temporal matching employing the start and ending frames; and τ is a threshold for the minimum spatial overlap using the corresponding bounding boxes. We use σ=τ=0.90 to ensure a high spatio-temporal overlap between candidates and ground-truth objects. As such systems are intentioned to help monitoring staff, pixel-level precision is not as important as detecting the event.

Then, we evaluate AOD performance for each configuration of the stages by accumulating the statistics for all sequences into Precision (*P*), Recall (*R*) and F-score (*F*) as:

(4) $$P=\frac{TP}{TP+FP}$$

(5) $$R=\frac{TP}{TP+FN}$$

(6) $$F=\frac{2\cdot P\cdot R}{P+R}$$

where TP, FP, and FN denote, respectively, correct, false, and missed detections. To apply this evaluation protocol, we use the ViPER-PE tool, which provides multiple metrics for comparing results and ground-truth data.

## 6. Results

This section evaluates the performance for each stage of the AOD system over all sequences in Table 5 where the abandoned objects lifespan is at least 30 s, i.e., 18 events. Instead of providing individual stage results, we study the impact of each stage on the AOD performance. Therefore, the evaluation of approaches for a particular stage considers fixing the other stages to a preferred best option.

### 6.1. Comparison of Foreground Segmentation Approaches

**Figure 10:**
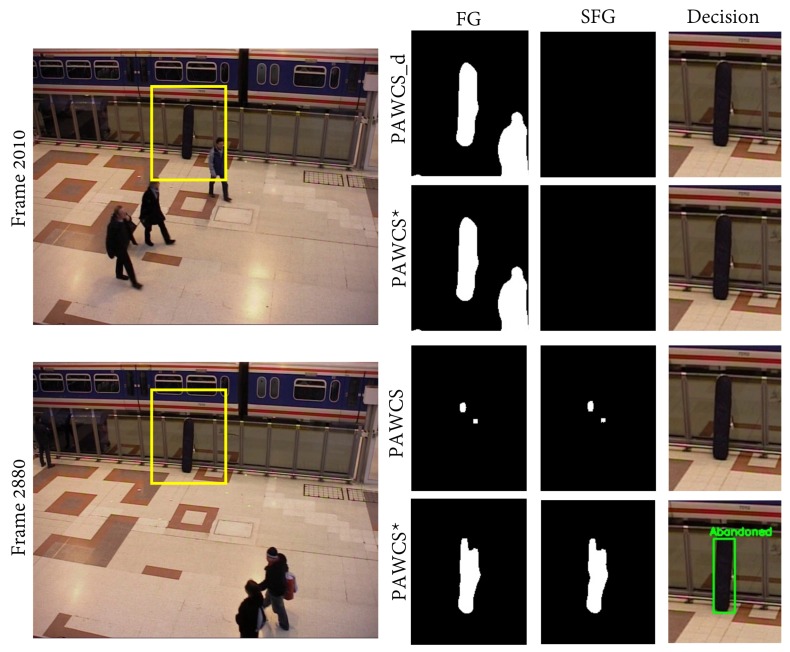
Columns from left to right show the frame image with event ROI marked in yellow, foreground mask, static foreground mask, and final decision. The first and second row show visual results at different stages of Frame 2010, when the object has just been left. Both default and tuned PAWCS detect the object as part of the foreground; however, for a further frame (third and fourth rows), only tuned PAWCS maintains the object detection, while default PAWCS misses the abandoned object.

Table 6 shows the configurations and results for evaluating the foreground segmentation performance of three BS approaches (MOG, KNN, and PAWCS). The other stages were fixed according to their performance provided by the respective authors. Bearing in mind that the stationary detection time was set to 30 s, the main explanation is related to the learning speed of the background subtraction algorithms. High (low) values imply that the background model quickly (slowly) absorbs the blobs resulting from foreground segmentation. Therefore, the stage for stationary foreground segmentation would not be able to detect any stationary information if blobs were absorbed faster than the stationary detection time. We have examined the performance of the three BS algorithms, firstly by using their default learning rate parameters, provided by the authors, and secondly by evaluating the best possible performance through manually tuning the learning parameters on each algorithm. To avoid background model updates for objects static during 30 s, we set the MOG learning rate α=0.00005, the KNN learning rate α=0.001, and for PAWCS, parameters $t_{0}=$ 10,000 and nSamples=20. Figure 10 shows the performance comparison between default and tuned PAWCS. One can observe that the default configuration of PAWCS absorbed the target object prematurely; consequently, it cannot be detected in subsequent stages.

**Figure 11:**
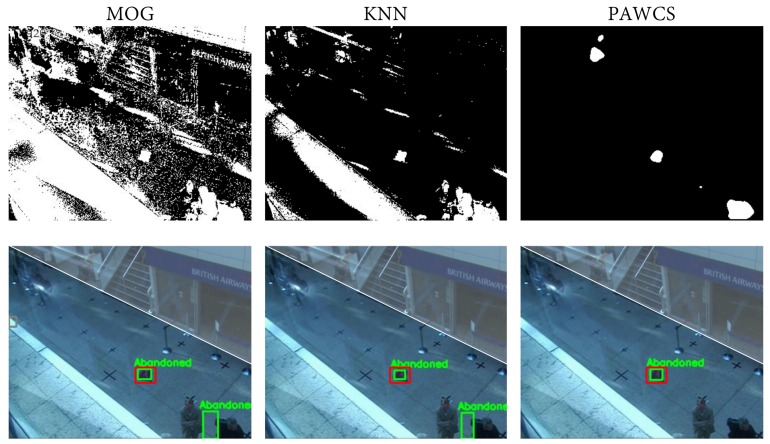
The first row shows the obtained foreground mask of the same frame of sequence PETS07 S7 C3 with the MOG, KNN, and PAWCS algorithm, respectively. The second row shows the correspondent abandoned discrimination. The ground-truth is marked in red; green shows the abandoned detections; and the non-interest region is colored in white. Significant differences between algorithms may be observed regarding the quality of the foreground masks. In this example. MOG and KNN are providing a ghost detection resulting in a false positive detection, which brings precision down.

Configurations #1–3 in Table 6 correspond to MOG, KNN, and PAWCS with the default configuration, and #4–6 refer to the same tuned algorithms. Tuning was done in such a way that the algorithms maintain the detections longer. As expected, default configurations provided inferior results since they are not aimed at maintaining foreground along time. This fact is clearly reflected for default MOG and KNN (#1–2), where no objects were detected since these algorithms absorbed foreground objects very quickly. Default PAWCS (#3) was able to maintain a larger number of detections; however, its tuned version (#6) performed better, as shown in Figure 10. Given the results, from now on, we will only focus on tuned algorithms. As might be expected, from the state-of-the-art change detection evaluations, such as CDNET [120], PAWCS globally performed better than KNN and MOG. The relative improvement with respect to MOG was 33%, whereas with regard to KNN, it was 7%. The reason MOG was providing such worse results is that it is very sensitive to illumination changes and shadows; thus, it provided more false positive detections, resulting in a lower precision measure; see Figure 11.

For subsequent experiments, we decided to keep the two FS algorithms with better overall measures (KNN and PAWCS).

### 6.2. Comparison of Static Foreground Segmentation Approaches

Table 7 shows the configurations and results to evaluate SFD performance. Note that each SFD approach needed to be specifically implemented for the selected BS approaches (KNN and PAWCS, according to the results in Table 6). From the table, one can observe, with the naked eye, that all PAWCS configurations (#6–10) overcame the KNN configurations (#1–5). For this reason, we will only focus on PAWCS for further experiments.

**Figure 12:**
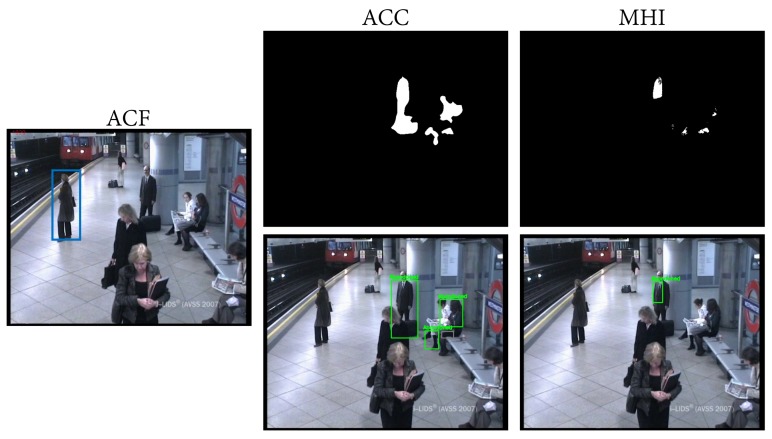
All images correspond to the same frame of the AVSS medium sequence. The image on the left shows in blue the detections provided by the ACF people detector. The top row illustrates the static foreground mask obtained with the ACC and MHI algorithms, respectively, and the bottom row shows their corresponding abandoned discrimination.

The previous PAWCS result (#6) was globally improved 7% by using the MHI algorithm (#3), due to a precision increase. As can be seen in Figure 12, MHI reduced false positive detections. A reasonable explanation for this is that the MHI algorithm incorporates motion information in the static foreground mask computation, unlike ACC, which only accumulates foreground mask. In this particular case, as sitting people are not completely still, MHI is able to filter them, while ACC wrongly incorporated them into the static foreground mask.

In light of the results, PAWCS and MHI were fixed for the next experiments.

### 6.3. Comparison of People Detection Approaches

The results of the people detection approaches’ comparison are shown in Table 8. It presents seven configurations with the implemented people detection algorithms whilst keeping fixed at the FS, SF,D and AD stages, PAWCS, MHI, and HG respectively. It is important to remark that regardless of the people detection performance, AOD recall can never be higher than the value reached in the previous stage (72%), since people detection filters the static foreground obtained at the SFD stage, i.e., it is not producing new abandoned object detections. However, such filtering can increase precision by removing stationary people.

**Figure 13:**
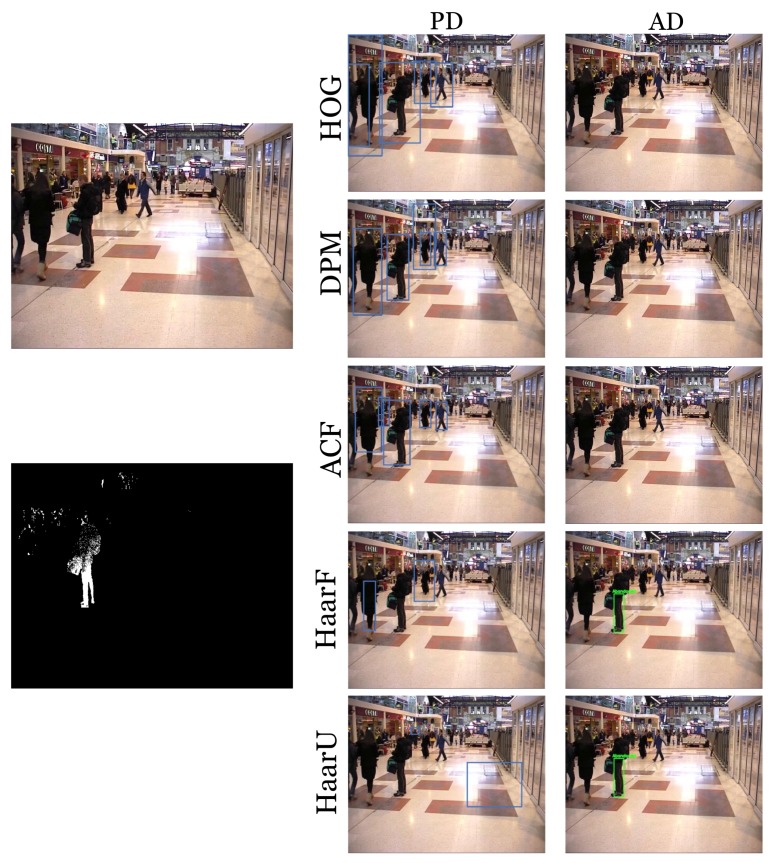
The first column shows the frame under consideration and its computed stationary foreground mask (DBM); the second column reports the visual results of the implemented people detector; and the third column presents the abandoned object discrimination of the system. It can be observed that the Haar-like feature classifier for full (HaarF) and upper body parts (HaarU) were not able to detect the standing stationary person; therefore, the system was mistakenly detecting him as an abandoned object.

Regarding the numeric results in Table 8, the DPM (#2), HaarF (#4), HaarU (#5), and YOLOv2 (#7) approaches maintained the previous ACF recall (#3). Regarding precision, the aforementioned algorithms barely modified it, except YOLOv2. Within the evaluated sequences, there were several videos containing people in different situations such as sitting, small people due to remoteness, or even partially occluded. Algorithms employing a more complex and efficient person model were able to detect more people in these scenarios, while the others missed those detections. Figure 13 exemplifies the differences between hand-crafted detectors and how their performance affects the results, in terms of precision. In this case, YOLOv2 is able to avoid any false positives caused by stationary people missed detections, leading to 100% precision. Due to its results, YOLOv2 was set at the people detection stage for the next experiments.

### 6.4. Comparison of Abandoned Discrimination Approaches

Abandoned discrimination configurations and their results are summarized in Table 9. This stage determined whether candidate objects were indeed abandoned objects. As this module filtered false candidates, it was not able to generate new ones, and therefore, as in PD stage, the previous recall (72%) could not be increased by improving this classification step.

**Figure 14:**
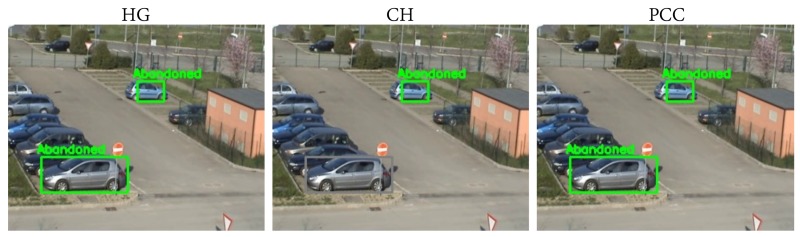
From left to right are shown the abandoned discrimination obtained with the HG, CH, and PCC algorithms for the same frame of the VISOR 00 sequence. HG and PCC correctly detected both cars as abandoned, while CH missed one of the detections (marked in grey) due to the wrong classification.

Comparing the results provided by the three classifiers, we can note that there was no difference between the performance of HG (#1) and PCC (#3); however, CH (#2) worsened the results by 7%. Each abandoned classification algorithm was based on a certain feature (color, edges, etc.) or even several ones; thus, these algorithms were highly dependent on the sequence itself. In the case under study, as seen in Section 4.4.1, HG employed gradient-based features; PCC combined color and gradient information; and CH was only focused on color. Differences in the performance of these algorithms are shown in Figure 14, where one can observe that CH was failing at classifying one of the cars as abandoned and classifying it as an illumination change, due to it only considering color information.

### 6.5. Computational Cost

We report the computational cost in terms of seconds per frame. The multi-configuration AOD system was implemented in C++ using the OpenCV Library (https://opencv.org/), programmed using a single thread without any GPU or parallel optimization. To report times, we used a standard desktop computer with 2.1 GHz and 4 GB RAM.

**Figure 15:**
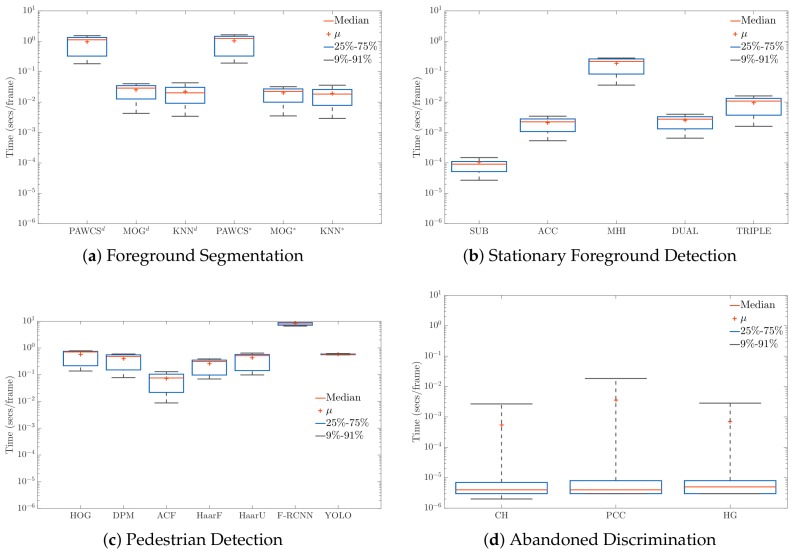
Computational cost analysis, in terms of seconds per frame, of every algorithm implemented at each stage: Foreground Segmentation (**a**), Stationary Foreground Detection (**b**), Pedestrian Detection (**c**) and Abandoned Discrimination (**d**).

Figure 15 shows a computational time comparison, by stage, of each algorithm. From the graphs, we can see that the FG and PD algorithms were the ones requiring the most computational time. It is important to remark that FG computation was performed every frame, while PD was only computed when an object was detected as stationary. For this reason, we can conclude that the choice of an efficient FG algorithm is crucial in AOD systems. For fair comparisons, note that GPU-capable algorithms in c++ OpenCV (F-RCNN and YOLO) have been only run on a CPU.

**Figure 16:**
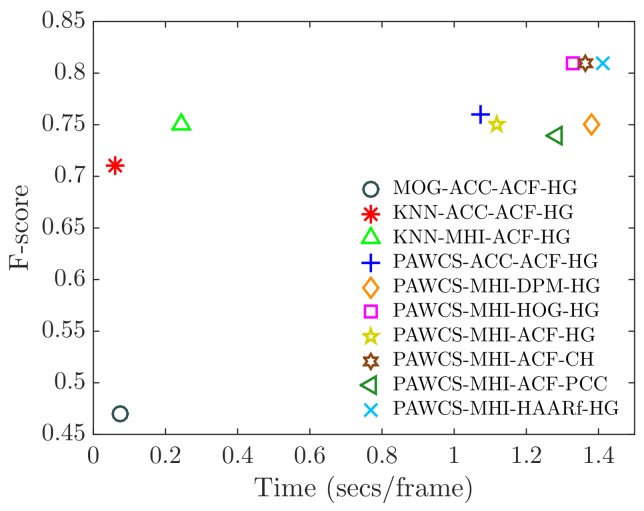
Relation between performance (F-score) and computational time (seconds per frame) of a selection of relevant configurations.

The relation between computational time and performance, in terms of F-score, is shown in Figure 16. Ten significant configurations have been chosen for the comparison. Note that F-RCNN and YOLOv2 have been excluded since they are optimally designed for GPU computing. From the graph, one can observe two separate groups. The MOG and KNN configurations took around 0.15 s to compute a frame, while PAWCS required around 1.2 s (eight-times slower). Again, one can observe the importance of having a fast and well-performing FS algorithm.

### 6.6. Validation

**Figure 17:**
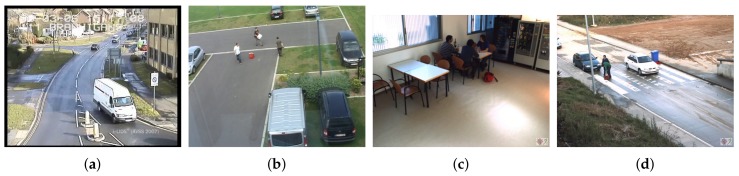
Sample frames from the (**a**) AVSS 2007 PV, (**b**) CANTATA C2, (**c**) HERMES Indoor, and (**d**) HERMES Outdoor datasets.

In this section, we perform a validation step by reporting the numerical results of AOD performance using the best configuration, derived from the previous study performed in Section 6 and also comparing it with a faster and simpler configuration. Both configurations were evaluated in video sequences from datasets not used in the previous evaluation. We selected eight sequences from the following public datasets: AVSS 2007 PV (http://www.eecs.qmul.ac.uk/~andrea/avss2007_d.html), CANTATA (http://www.multitel.be/~va/cantata/LeftObject/), HERMESIndoor (http://iselab.cvc.uab.es/silverage.php?q=indoor-cams), and HERMES Outdoor (http://iselab.cvc.uab.es/silverage.php?q=outdoor-cams). Figure 17 shows sample frames of them, and also, some information can be found in Table 4. AVSS 2007 PV is a parked vehicle scenario providing very noisy and low-quality images and also presenting a high density of objects. We evaluated the sequence called AVSS PV Eval. CANTATA is an outdoor left objects scenario. We selected three sequences: C2_3, C2_9, and C2_17, presenting strong illumination changes and also removed objects. From HERMES Indoor and HERMES Outdoor, we evaluated sequences C1, C2, C3, and C4, respectively. In short, performance was validated in a varied set of eight sequences presenting different challenges. The results per dataset and the average results are depicted in Table 10. Focusing on the average results, the best previous configuration (PAWCS, MHI, YOLOv2, and HG) reached a 0.84 F-score, while in validation, the F-score was 0.81. Hence, one can state that the selected algorithms were able to keep their behavior in different challenging scenarios. In addition, we show the validation results with a simpler and faster selection of algorithms (KNN, ACC, ACF, and HG), which, as expected, was much less robust; however, conversely, its computational time was one order of magnitude lower, as seen in the previous section.

## 7. Conclusions

Automatic event detection is a fundamental, but challenging issue in the field of video-surveillance. More precisely, Abandoned Object Detection (AOD) has attracted huge interest in the last few years for monitoring potentially risky public and private places.

In this paper, we state the framework employed by AOD systems, and we extensively go over the state-of-the-art approaches and their respective stages: moving and stationary foreground detection, people detection, and abandonment verification. We also organize and perform experimental comparisons of traditional and recent approaches over a varied set of sequences from public databases through a multi-configuration system. The proposed system allows selecting algorithms out of a selection for each stage; thus, a large range of AOD systems can be compared for a deep study of the trade-off between accuracy and computational cost. This is a key contribution that has not provided by any previous survey.

From the experimental comparison, in Section 6, one can draw some conclusions. Although every stage was important in the AOD procedures, each of them has a different impact on the final results. AOD is a sequential operation where each stage operates on the output of the previous one, and for this reason, foreground segmentation is the first and most critical stage. One of the main challenges for FS algorithms is camouflage, and as we have been able to verify, it is still an open challenge for state-of-the-art techniques. We also came to the conclusion that there exists a high dependency between the FS learning rate and the consequent further processing. Final recall also relies on the capability to determine whether the foreground is stationary or not. It is a challenging task if the target is occluded; thus, adding motion information is an improvement to be considered. Regarding people detection, the findings of this study support the idea that hand-crafted feature-based approaches are less efficient, in complex scenarios, than recent deep learning methods. Finally, it is important to consider an abandoned discrimination approach as comprehensively as possible. As future work, we will consider improving the experimental validation by creating a large-scale dataset for objects with different static times, as well as extending the multi-configuration system with recent advances.

## Figures and Tables

**Table 1:** Comparison with related surveys for the video-surveillance domain.

Reference	Topic Coverage for Abandoned Object Detection							
	Moving Foreground Segmentation	Stationary Object Detection	People Detection	Behavior Recognition	Abandoned Classification	Dataset Analysis	Experimental Validation	Software Provided
[3]	✓			✓				
[6]		✓					✓	
[8]			✓				✓	
[12]				✓		✓		
[14]	✓			✓				
[16]	✓							
[10]	✓			✓		✓		
[11]				✓		✓		
[4]	✓					✓		
[17]				✓			✓	
[9]			✓				✓	
[7]	✓	✓						
[15]	✓			✓		✓		
[18]	✓			✓				
[13]				✓		✓		
[5]	✓					✓		
Proposed	✓	✓	✓	✓	✓	✓	✓	✓

**Table 2:** Most relevant stationary foreground detection algorithms.

Algorithm	Type	Static Foreground Refinements			
		Filtering	Object Model	Alarm Type	Object Owner
[56]	Single FG mask	✓		✓	
[57]		✓			
[58]			✓	✓	✓
[59]	Multiple FG mask				
[46]		✓		✓	
[60]					✓
[61]	Model stability	✓	✓		
[47]		✓	✓		
[62]		✓	✓	✓	✓

**Table 3:** People detection categories’ robustness summary.

Category	Partial Occlusions	Pose Variations
Motion-based	No	Depending on the model
Holistic Appearance	No	No
Part-based Appearance	Yes	No
Hybrid	Yes	Depending on the model

**Table 4:** Abandoned Object Detection (AOD) datasets available. Key: I = Illumination changes/shadows; R = Remoteness/small objects; P = stationary People; O = Occlusions; LR = Low Resolution; RO = Removed Objects.

Dataset	# of Sequences	Avg Length (min)	Scenario	Challenges
ABODA (http://imp.iis.sinica.edu.tw/ABODA/)	11	1	Indoor/Outdoor	I, R, P, O
AVSSAB2007 (http://www.eecs.qmul.ac.uk/~andrea/avss2007_d.html)	3	3.5	Railway Station	I, R, P, O
AVSS PV2007 (http://www.eecs.qmul.ac.uk/~andrea/avss2007_d.html)	4	3	Road Way	I, P, O, RO
CANDELA (http://www.multitel.be/image/research-development/research-projects/candela/abandon-scenario.php)	16	0.5	Indoor	R, O, LR
CANTATA (http://www.multitel.be/~va/cantata/LeftObject/)	20	2	Outdoor	I, RO
CAVIAR (http://groups.inf.ed.ac.uk/vision/CAVIAR/CAVIARDATA1/)	5	1	Terrace	I, R, LR, RO
ETISEOBC (https://www-sop.inria.fr/orion/ETISEO/download.htm#video_data)	6	2	Indoor	I, R, LR
ETISEO MO (https://www-sop.inria.fr/orion/ETISEO/download.htm#video_data)	9	3	Subway	I, R, O, LR
HERMESIndoor (http://iselab.cvc.uab.es/silverage.php?q=indoor-cams)	4	2	Indoor	P, O
HERMES Outdoor (http://iselab.cvc.uab.es/silverage.php?q=outdoor-cams)	4	2	Road Way	P, O
PETS2006 (http://www.cvg.reading.ac.uk/PETS2006/data.html)	28	1.5	Railway Station	I, R, P, O
PETS 2007 (http://www.cvg.reading.ac.uk/PETS2007/data.html)	32	2.5	Railway Station	I, P, O, RO
VISORAB (http://imagelab.ing.unimore.it/visor/video_videosInCategory.asp?idcategory=14)	9	0.16	Indoor	-
VISOR SV (http://imagelab.ing.unimore.it/visor/video_videosInCategory.asp?idcategory=12)	4	0.16	Outdoor	I,R

**Table 5:** Summary of annotated abandoned objects.

ID	# of AO	Sequence	Lifespan	Unattended
1	2	VISOR 00	64/33	✓
2	1	VISOR 01	70	✓
3	2	VISOR 02	74/46	✓
4	1	VISOR 03	56	✓
5	1	AVSS07 E	64	✓
6	1	AVSS07 M	69	✓
7	1	AVSS07 H	90	✓
8	1	PETS06 S1 C1	34	✓
9	1	PETS06 S1 C3	34	✓
10	1	PETS06 S1 C4	34	✓
11	1	PETS06 S4 C1	73	-
12	1	PETS06 S4 C3	73	-
13	1	PETS06 S4 C4	73	-
14	1	PETS06 S5 C1	50	✓
15	1	PETS06 S5 C3	50	✓
16	1	PETS06 S5 C4	50	✓
17	1	PETS07 S7 C3	35	✓
18	1	PETS07 S8 C3	40	✓
19	1	ABODA 01	23	✓
20	1	ABODA 03	29	✓
21	1	ABODA 09	45	✓

**Table 6:** Results comparing foreground segmentation approaches for AOD performance. Bold indicates the best results. Key: FS = Foreground Segmentation. SFD = Stationary Foreground Detection. PD = Pedestrian Detection. AD = Abandoned Discrimination. *P* = Precision. *R* = Recall. *F* = F-score. ACF = Aggregated Channel Feature. HG = High Gradients.

Stage Configuration					AOD Performance		
#	FS	SFD	PD	AD	*P*	*R*	*F*
1	MOG2_d	ACC	ACF	HG	-	0.00	-
2	KNN_d				-	0.00	-
3	PAWCS_d				0.58	0.61	0.59
4	MOG2 *				0.38	0.61	0.47
5	KNN *				0.75	0.67	0.71
6	PAWCS *				**0.81**	**0.72**	**0.76**

**Table 7:** Results comparing stationary foreground segmentation approaches for AOD performance. Bold indicates the best results. Key: FS = Foreground Segmentation. SFD = Stationary Foreground Detection. PD = Pedestrian Detection. AD = Abandoned Discrimination. *P* = Precision. *R* = Recall. *F* = F-score.

Stage Configuration					AOD Performance		
#	FS	SFD	PD	AD	*P*	*R*	*F*
1	KNN *	ACC	ACF	HG	0.75	0.67	0.71
2		SUB			0.58	0.61	0.59
3		MHI			0.86	0.67	0.75
4		DBM			0.73	0.61	0.67
5		TBM			0.61	0.61	0.61
6	PAWCS *	ACC	ACF	HG	0.81	0.72	0.76
7		SUB			0.86	0.67	0.75
8		MHI			**0.93**	**0.72**	**0.81**
9		DBM			0.80	0.67	0.73
10		TBM			0.85	0.61	0.71

**Table 8:** Results comparing people detectors for AOD performance. Bold indicates the best results. Key: FS = Foreground Segmentation. SFD = Stationary Foreground Detection. PD = Pedestrian Detection. AD = Abandoned Discrimination. *P* = Precision. *R* = Recall. *F* = F-score. YOLO = You Only Look Once.

Stage Configuration					AOD Performance		
#	FS	SFD	PD	AD	*P*	*R*	*F*
1	PAWCS *	MHI	HOG	HG	0.86	0.67	0.75
2			DPM		0.93	**0.72**	0.81
3			ACF		0.93	**0.72**	0.81
4			HaarF		0.76	**0.72**	0.74
5			HaarU		0.72	**0.72**	0.72
6			F-RCNN		0.92	0.67	0.77
7			YOLOv2		**1**	**0.72**	**0.84**

**Table 9:** Results comparing the approaches of abandoned discrimination for AOD performance. Bold indicates the best results. Key: FS = Foreground Segmentation. SFD = Stationary Foreground Detection. PD = Pedestrian Detection. AD = Abandoned Discrimination. *P* = Precision. *R* = Recall. *F* = F-score. CH = Color Histograms.

Stage Configuration					AOD Performance		
#	FS	SFD	PD	AD	*P*	*R*	*F*
1	PAWCS *	MHI	YOLOv2	HG	**1**	**0.72**	**0.84**
2				CH	1	0.67	0.81
3				PCC	**1**	**0.72**	**0.84**

**Table 10:** Results comparing the approaches of abandoned discrimination for AOD performance. Bold indicates average results. Key: FS = Foreground Segmentation. SFD = Stationary Foreground Detection. PD = Pedestrian Detection. AD = Abandoned Discrimination. *P* = Precision. *R* = Recall. *F* = F-score.

AOD Performance																			
Stage Configuration					Average			AVSS PV			CANTATA			HERMES I			HERMES O		
#	BS	SFD	PD	AD	P	R	F	P	R	F	P	R	F	P	R	F	*P*	*R*	*F*
1	PAWCS *	MHI	YOLOv2	HG	**0.73**	**0.92**	**0.81**	0.67	1	0.8	1	0.83	0.91	0.5	1	0.67	0.67	1	0.8
2	KNN *	ACC	ACF	HG	**0.26**	**0.92**	**0.41**	0.12	1	0.21	0.36	0.83	0.5	0.22	1	0.36	1	1	1
